# Challenges in attaining universal health coverage: empirical findings from Rashtriya Swashthya Bima Yojana in Chhattisgarh

**DOI:** 10.1186/1753-6561-6-S5-O12

**Published:** 2012-09-28

**Authors:** Sulakshana Nandi, Rajib Dasgupta, Kanica Kanungo, Madhurima Nundy, Ganapathy Murugan

**Affiliations:** 1 Public Health Resource Network, Delhi, India; 2 Jawaharalal Nehru University, Delhi, India

## Introduction

The Rashtriya Swasthya Bima Yojana (RSBY) is a financial protection scheme in India. It is aimed at protecting families below poverty line (BPL) against catastrophic health expenses. It also seeks to increase their access to healthcare and expand the choice of providers.

We undertook a series of studies in Chhattisgarh to examine: a) the key issues in the design and implementation of the scheme and b) ask if the objectives of the scheme are being fulfilled particularly for the marginalized communities in underserved areas. This is significant as the scheme is being promoted as the model and vehicle for universal access to healthcare.

## Methods

This paper draws evidence from three studies undertaken in Chhattisgarh in 2011-12. We undertook a quantitative study on enrolment in the RSBY scheme in 270 villages (18 Districts) along with People’s Health Movement, Chhattisgarh. Data were collected through a village level questionnaire.

We also did a qualitative study to understand the issues around the design of the scheme through mapping the providers’ perspectives. Providers were interviewed in three districts. They were drawn from private for-profit (small 10-20 bedded nursing homes and multi-specialty corporate hospitals), public (medical college, district and sub-district hospitals) and not-for-profit (low-cost and Christian missionary) institutions. Interviews were also held with state-level administrators.

A quantitative study on access of Particularly Vulnerable Tribal Groups (PTGs) to health and nutrition services was undertaken by the research team along with other NGOs. A household questionnaire was administered to 1200 families belonging to these groups.

## Results

Our study results show that there had been very low enrolment and usage in remote villages and among vulnerable communities. Instances of denial, lack of transparency and sharing of information with beneficiaries and high out of pocket expenditure were the common findings emerged from the quantitative surveys. Only 32% of the PTG families were enrolled, of whom only 4% had used the card.

Private empanelled facilities were providing narrow and selective range of services. They picked and chose more profitable conditions/packages though experiencing an increase in case load.

Public hospitals reported decline in the number of patients. The public hospitals were unable to compete with private hospitals in better-off areas but reported higher numbers of beneficiaries in tribal blocks. The public hospitals were treating common medical conditions with few surgical conditions/procedures.

We observed that package rates were not sufficient for complications requiring long stay or expensive medication. Both public and private providers were performing few high-end procedures. Not-for-profit hospitals, on the other hand, provided a relatively large range of services (surgeries, orthopaedic procedures, chemotherapy) and reported increase in case loads. They undertook some cost-cutting measures, though without compromising on quality.

We observed that settlement/rejection of claims seemed *ad hoc* and providers adopted ‘defensive’ (sometimes corrupt) practices against losses.

## Discussion

The study shows that RSBY is far from achieving it objectives. Most vulnerable communities are being left out from this financial protection scheme. There is no guarantee of services for the poor. Conversely, private nursing homes benefit from this scheme with increased turnover and income as they selectively choose to treat medical conditions and patients. The public health system is unable to compete and is weakening. Costs of care for medical conditions are being artificially inflated.

There is need for a strong monitoring and grievance redressal mechanism, including transparency during empanelment. Time-bound settlement of claims needs to be ensured through penalties for delays. System for referral and complications need to be evolved and cost for high end packages needs to be revised and made more realistic.

## Competing interests

Authors declare that they have no conflict of interest.

## Funding statement

The study on design issues was funded by the ICICI Foundation for Inclusive Growth – Centre for Child health and Nutrition (IFIG-CCHN).

